# *HPDL* Variant Type Correlates With Clinical Disease Onset and Severity

**DOI:** 10.1002/acn3.70047

**Published:** 2025-05-14

**Authors:** Eun Hye Lee, Olivia Kim‐Mcmanus, Jennifer H. Yang, Richard Haas, Maha S. Zaki, Ghada M. H. Abdel‐Salam, Yuji Nakamura, Mohamed S. Abdel‐Hamind, Darius Ebrahimi‐Fakhari, Julian E. Alecu, Nicola Brunetti‐Pierri, Varunvenkat M. Srinivasan, Vykuntaraju K. Gowda, Stephanie Gross, Yasemin Alanay, Paria Najarzadeh Totbati, Manya Yadavilli, Liana Friedman, Naomi Meave Ojeda, Joseph G. Gleeson

**Affiliations:** ^1^ Rady Children's Institute for Genomic Medicine San Diego California USA; ^2^ Department of Pediatrics, College of Medicine Kyung Hee University Seoul Korea; ^3^ Department of Neurosciences and Pediatrics University of California San Diego California USA; ^4^ Human Genetics and Genome Research Division, Clinical Genetics Department National Research Centre Cairo Egypt; ^5^ Human Genetics and Genome Research Division, Medical Molecular Genetic Department National Research Centre Cairo Egypt; ^6^ Department of Neurology, F.M. Kirby Neurobiology Center, Boston Children's Hospital Harvard Medical School Boston Massachusetts USA; ^7^ Friedrich‐Alexander‐University Erlangen‐Nuremberg Erlangen Germany; ^8^ Telethon Institute of Genetics and Medicine Naples Italy; ^9^ Department of Translational Medicine Federico II University of Naples Naples Italy; ^10^ Department of Pediatric Neurology Indira Gandhi Institute of Child Health Bangalore India; ^11^ Department of Pediatric Neurology, Social Pediatrics and Epileptology Center for Pediatrics and Adolescent Medicine at the University Hospital Giessen and Marburg GmbH Marburg Germany; ^12^ Pediatric Genetics Unit, Department of Pediatrics, School of Medicine Acibadem Mehmet Ali Aydinlar University Istanbul Turkey; ^13^ Rare Diseases and Orphan Drugs Application and Research Center, ACURARE Acıbadem University Istanbul Turkey

**Keywords:** 4‐hydroxyphenylpyruvate dioxygenase‐like protein, encephalopathy, hereditary spastic paraplegia, HPDL, mitochondria

## Abstract

**Objective:**

Recently, a mitochondrial encephalopathy due to biallelic *HPDL* variants was described, associated with a broad range of clinical manifestations ranging from severe, infantile‐onset neurodegeneration to adolescence‐onset hereditary spastic paraplegia. HPDL converts 4‐hydroxyphenylpyruvate acid (4‐HPPA) into 4‐hydroxymandelate (4‐HMA), necessary for the synthesis of the mitochondrial electron transporter CoQ10. This suggests a possible bypass of the metabolic block by 4‐HMA treatment; however, genotype–phenotype correlations are lacking.

**Methods:**

We established an HPDL Patient Registry to prepare for a future clinical trial. Here we report the clinical features of 13 enrolled participants and compare them with 86 previously reported patients. We establish three major clinical classes: severe, intermediate, and mild, presenting onset in early infancy, childhood, and adolescence, respectively. The biallelic genotypes were classified into truncating/truncating, truncating/missense, and missense/missense variants, mapped onto the predicted 3D protein structure, and correlated with severity.

**Results:**

Patients with biallelic truncating variants presented with severe phenotypes and earlier ages of onset. Missense variants were often associated with milder phenotypes, except those with variants predominantly located in or near the VOC2 domain containing iron‐binding sites or the C‐terminus, which had more severe phenotypes. In addition, p.Met1? variants were also correlated with more severe phenotypes.

**Interpretation:**

This study demonstrates the correlation of age of onset and disease severity with genotype for *HPDL‐*related conditions. Patients with truncating variants and specific missense variants correlated with severe, early‐onset features, whereas the presence of at least one missense variant located outside of the iron‐binding sites correlated with milder presentations.

**Trial Registration:**

Clinicaltrials.gov HPDL registry: https://clinicaltrials.gov/study/NCT05848271

## 1 Introduction

Mitochondrial diseases are a group of complex metabolic disorders caused by genetic defects that predominantly affect oxidative phosphorylation, mitochondrial maintenance, mtDNA translation, and iron–sulfur biogenesis. These conditions present significant challenges for both diagnosis and treatment, due to a diversity of presentations, clinical courses, and a lack of effective medications, with an estimated prevalence of approximately 1:4000 individuals [1].

Causes of mitochondrial diseases include mutations in both mitochondrial and nuclear genomes. Establishing a genetic diagnosis for most patients, particularly those with nuclear gene mutations, was challenging in the past. However, next generation sequencing has opened the doors for identifying genes impacting mitochondrial function, with pathogenic variants identified in over 300 genes to date [2]. Identifying specific genetic defects holds great promise for both identifying disease mechanisms as well as developing new therapies.

**FIGURE 1:**
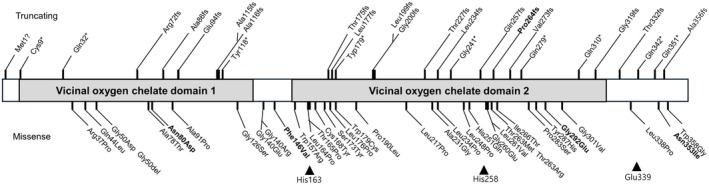
Structure of HPDL protein with truncating and missense variants. Truncating variants are annotated above the HPDL protein structure, while missense variants are below. Bold indicated the new variants from our cohort. The truncating and missense variants are widely spread on a whole protein. Arrowheads below reference iron binding residues.

Recently, biallelic variants in *HPDL* (4‐hydroxyphenylpyruvate dioxygenase‐like) were identified as causes of a rare mitochondrial encephalopathy (ME), associated with a broad range of clinical manifestations, from severe, infantile‐onset neurodegeneration to pure or complex spastic paraplegia. *HPDL* is a nuclear gene located on chromosome 1p34.1 and consists of a single exon. It encodes a 371 amino acid protein, HPDL, which is an intermembrane mitochondrial protein. The HPDL protein contains a predicted mitochondrial targeting sequence (MTS), two vicinal oxygen chelate (VOC) domains from amino acids 7–135 and 160–328, and three regions involved in iron (Fe)‐binding (Figure 1). In 2021, we reported 17 patients presenting with infantile‐onset neurodevelopmental disorder characterized by progressive spasticity and brain white matter abnormalities, which we termed NEDSWMA (MIM#619026). Similar reports identified *HPDL* variants in patients with a broader phenotypic spectrum with severe to mild disease, including a form of adolescent‐onset hereditary spastic paraparesis (SPG83; MIM# 619027) [3, 4, 5]. The function of HPDL remained enigmatic until recently when it was found to mediate the oxidation of a tyrosine metabolite termed 4‐hydroxyphenylpyruvate acid (4‐HPPA) to 4‐hydroxymandelate (4‐HMA), required for the synthesis of the CoQ10 headgroup [6]. These links suggest treatment of HPDL might be possible with dietary supplementation with small molecules like 4‐HMA to bypass the metabolic block.

Here we investigate genotype–phenotype correlations in HPDL‐related disease and lay the groundwork for prospective natural history studies to evaluate potential therapies.

## 2 Materials and Methods

The institutional review board (IRB) at the University of California–San Diego (UCSD) approved this study (IRB‐140028). Written informed consent was obtained from the parents or guardians of all participants. We conducted a systematic PubMed search for *HPDL*, which identified nine relevant publications comprising 86 patients, reviewing the clinical findings and genotype information, in comparison with newly enrolled patients [3, 4, 5, 7, 8, 9, 10, 11, 12]. Simultaneously, we established an HPDL patient registry, which has been enrolling patients since March 2023. We recruited by contacting authors and posting entries to the ClinVar website, the authors of previous HPDL papers, and clinical sequencing providers. Referred patients worldwide who consented to participate were enrolled in this genotype–phenotype study.

Genotypes were classified based on zygosity and specific variant consequences. Stop codon, frameshift, and start loss variants were categorized as truncating variants due to their presumed impact on protein function. Missense variants were considered non‐truncating. Clinical features assessed included age at onset, initial symptoms, presence of seizures, spasticity, encephalopathy, disease progression, movement disorders, and intellectual disability. Brain imaging findings were analyzed for cortical atrophy, white matter abnormalities, and corpus callosum abnormalitieshypoplasia.

Association with each genotype was examined. Missense variants were computationally annotated using data from SIFT, CAD, and Polyphen2 via the Ensembl Variant Effect Predictor online interface [13]. Protein structure changes were visualized and modeled using the PyMOL Molecular Graphics System (Version 2.3.2; Schrödinger LLC, New York, USA). Statistical analyses were conducted using SPSS Version 28.0. The chi‐square test was utilized to compare differences between groups, and survival rates were estimated by the Kaplan–Meier method. The log‐rank test was used to assess differences between groups. A *p*‐value < 0.05 was considered statistically significant after correcting for multiple comparisons.

## 3 Results

### 3.1 Identification of New Patients and Pathogenic Variants

From our registry, we identified 13 patients (and their parents) with *HPDL* pathogenic variants who provided informed consent, allowing us to collect clinical and genetic information. By integrating these 13 new patients and 86 patients from the literature [3, 4, 5, 7, 8, 9, 10, 11, 12], a total of 99 patients with 63 distinct pathogenic *HPDL* variants were considered, including five novel variants we report in this study.

### 3.2 Clinical Features of the Three Phenotypic Groups

We conducted a thorough review of a total of 99 patients with *HPDL*‐related disease, including 13 new patients from our cohort. Based on clinical presentation and disease course, patients were classified into three phenotypic groups: (1) developmental epileptic encephalopathy categorized as severe, (2) mild or normal intelligence exhibiting solely motor spasticity categorized as mild, and (3) intermediate severity as those falling between these two groups. Detailed clinical and genetic profiles of the 13 newly enrolled patients are provided in Table S1. Among the total 99 individuals, 48 had the severe phenotype, 29 had the intermediate phenotype, and 22 had the mild phenotype (Table 1).

**TABLE 1:** Demographic and clinical features of *HPDL*‐related disease.

	Severe	Intermediate	Mild
	*N* = 48	*N* = 29	*N* = 22
Age on onset (months, mean ± SD)	2.8 ± 3.3	35.2 ± 26.9	157.2 ± 29.9
Sex (male:female)	28:20	17:12	17:5
Death	12 (25%)	0	0
Symptom at onset			
Seizure	39	1	0
Motor developmental delay	6	13	0
Developmental regression	0	2	0
Global developmental delay	1	0	0
Encephalopathy	1	0	0
Gait instability	0	13	20
Microcephaly	27/30	3/13	0/16
Intellectual disability/GDD	48/48	20/29	6/22
Spasticity	47/48	29/29	22/22
Seizure	46/48	4/29	0
Intermittent deterioration	8/48	1/29	0
Oculomotor abnormalities	14/25	14/21	1/10
Cortical atrophy	28/44	6/24	0/17
Corpus callosum hypoplasia	34/44	9/24	2/17
White matter abnormalities	40/44	5/24	0/17

Abbreviation: GDD, global developmental delay.

Severe patients presented at an average onset age of 2.8 ± 3.3 months, with seizures and global developmental delay (GDD) observed in 46/48 (95.8%) and 48/48 (100%) of cases within this category, respectively. Severe patients showed microcephaly (< 2 SD) in 27/30 (90%), along with a range of MRI findings that included white matter abnormalities in 40/44 (90.1%), corpus callosum hypoplasia in 34/44 (77.2%), and cortical atrophy in 28/44 (63.6%). Intermediate patients typically presented at late infancy to mid‐childhood with a mean age of onset of 35.2 months and were characterized by motor developmental delay and predominantly lower limb spasticity; in two of these patients, symptoms began with developmental regression, where patients initially exhibited typical development followed by loss of skills by the age of 4 years, and 3 years 2 months, respectively. The former patient had biallelic truncating variants (c.1024C>T;p.Gln342* homozygous) [5]. The latter patient had biallelic missense variants (c.875G>A;p.Gly292Glu homozygous), and was completely typical till the age of 3 years 2 months and then presented difficult speaking, along with unsteady gait, epilepsy, and reduced cognitive function (Case 11 from Table S1). In the intermediate group, 4 of 29 (13.8%) of patients experienced seizures, which was significantly lower in frequency compared to the severe phenotype group (*p* < 0.001; chi‐square analysis). Intermediate patients displayed intellectual disability in 20/29 (69.0%), and corpus callosum hypoplasia, cortical atrophy and white matter abnormalities in 9/24 (37.5%), 6/24 (25.0%), and 5/24 (20.8%), respectively. Patients with the mild phenotype typically manifested at a mean age of onset of 13 years and 1 month, predominantly presenting gait abnormalities or frequent falls. Most mild patients showed normal MRI findings, although 2 of 17 patients (11.8%) exhibited mild corpus callosum hypoplasia. No patients in this group had severe intellectual disability or epilepsy, indicating distinct clinical manifestations from the severe or intermediate phenotypes.

**FIGURE 2:**
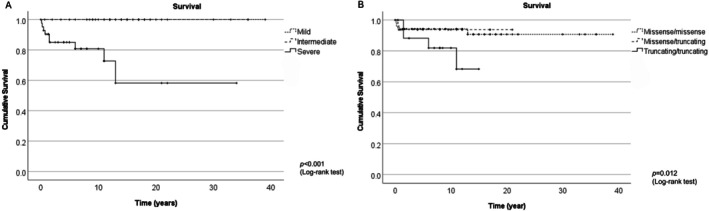
(A) Survival rate in different phenotypes. (B) Survival rate based on genotypes. Severe phenotype and truncating/truncating variants correlated with lower survival rates (*p* < 0.001, *p* = 0.012, respectively).

The severe phenotype was often associated with a neonatal or infantile presentation with onset occurring before 12 months of age, leading to death. Notably, 12 of 48 (25.0%) patients with the severe phenotype have died from *HPDL*‐related disease (Figure 2). Causes of mortality were attributed to seizures (9 cases), pneumonia (2 cases), and encephalopathy (1 case) [3, 4, 11]. In contrast, no deaths were observed among patients with intermediate or mild phenotypes.

### 3.3 Genotype–Phenotype Correlation in *HPDL* ‐Related Disease

The most prevalent pathogenic variant in this study was c.149G>A (p.Gly50Asp). Among the 99 patients, 14 were found to be homozygous for the c.149G>A (p.Gly50Asp) variant, which showed a clear association with a mild phenotype (Table S2). Eleven out of 14 (78.5%) had onset after the age of 10 years, while three showed onset before the age of 10 years. Excluding one patient who had onset at the age of 3 years with delayed motor development, the remaining 13 patients exhibited the mild phenotype. These individuals presented with gait abnormalities such as lower leg spasticity and frequent falls, without epilepsy or severe intellectual disability. Ten underwent brain MRI, nine of which showed normal findings and one patient had cerebellar atrophy. Additionally, three siblings were identified as compound heterozygous for p.Gly50Asp (c.140C>G) in association with the variant p.Tyr118* (c.353dupA) and exhibited delayed walking and progressive spasticity at the age of 2 years, thus classified as intermediate phenotype.

Three patients from two families had homozygous pathogenic variants in c.3G>C (p.Met1?). The age of onset in these three homozygous patients was 0.5, 4, and 7 months respectively, with all presenting severe GDD, seizures, and lethargy. MRI revealed severe cortical atrophy, corpus callosal hypoplasia, and white matter defects, indicating a severe phenotype associated with this pathogenic variant.

**FIGURE 3:**
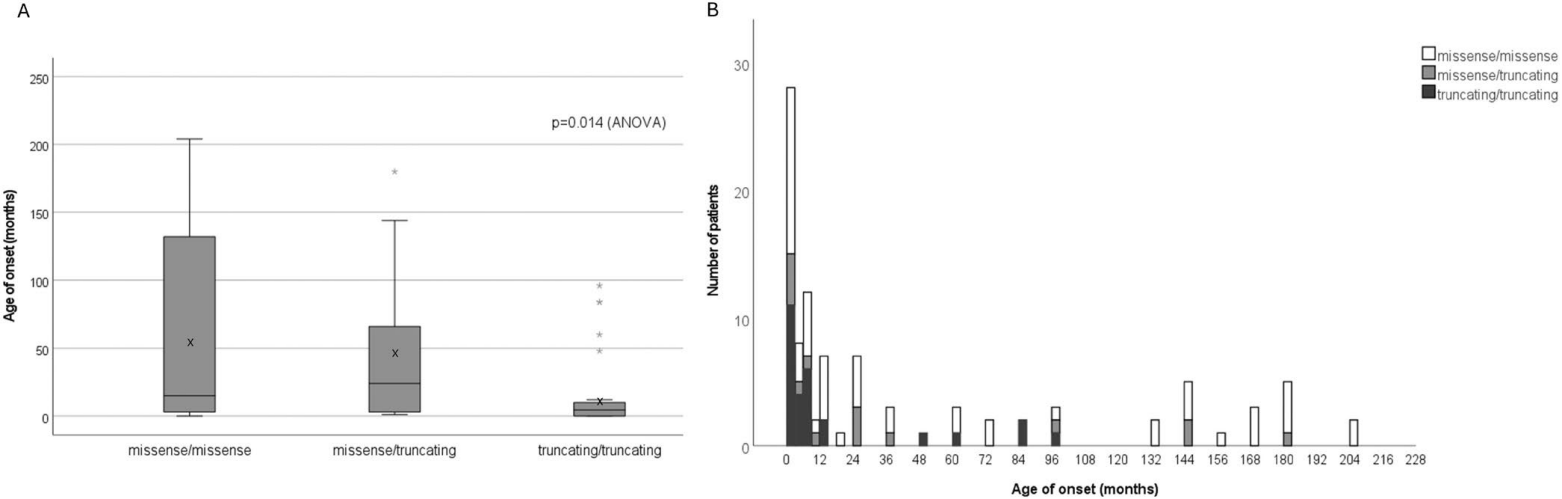
(A) The mean ages of onset of missense/missense, missense/truncating, and truncating/truncating variants were 58.3, 46.5, and 16.1 months, respectively (*p* = 0.014). Each star indicates mean onset age. (B) Truncating/truncating variants associated with early age onset, especially onset under 12 months. Some missense/missense variants also correlated with a very early onset severe phenotype.

Within the truncating/truncating group, 75.0% exhibited a severe phenotype, while none of the individuals with truncating/truncating variants exhibited a mild phenotype (Table S3). The missense/missense variants group showed an even distribution in clinical severity phenotypes, with 36.4% displaying a severe phenotype. Comparison of age of onset among the three groups revealed that the truncating/truncating group presented earliest (mean 16.1 ± 28.9 months), and the missense/missense group presented later in life (mean 58.3 ± 71.2 months), and the missense/truncating group fell between (mean 46.5 ± 62.0 months) (*p* = 0.014; ANOVA, Figure 3). Case 9 in our cohort (Table S1) with truncating/truncating variants developed rapidly progressing spastic gait and frequent falls at the age of 8 years. His parents informed us that his two younger siblings had died from severe encephalopathy due to the same variants at early infancy, further supporting the association between severe clinical manifestations and truncating variants.

### 3.4 Structural Analysis of Missense Variants

**FIGURE 4:**
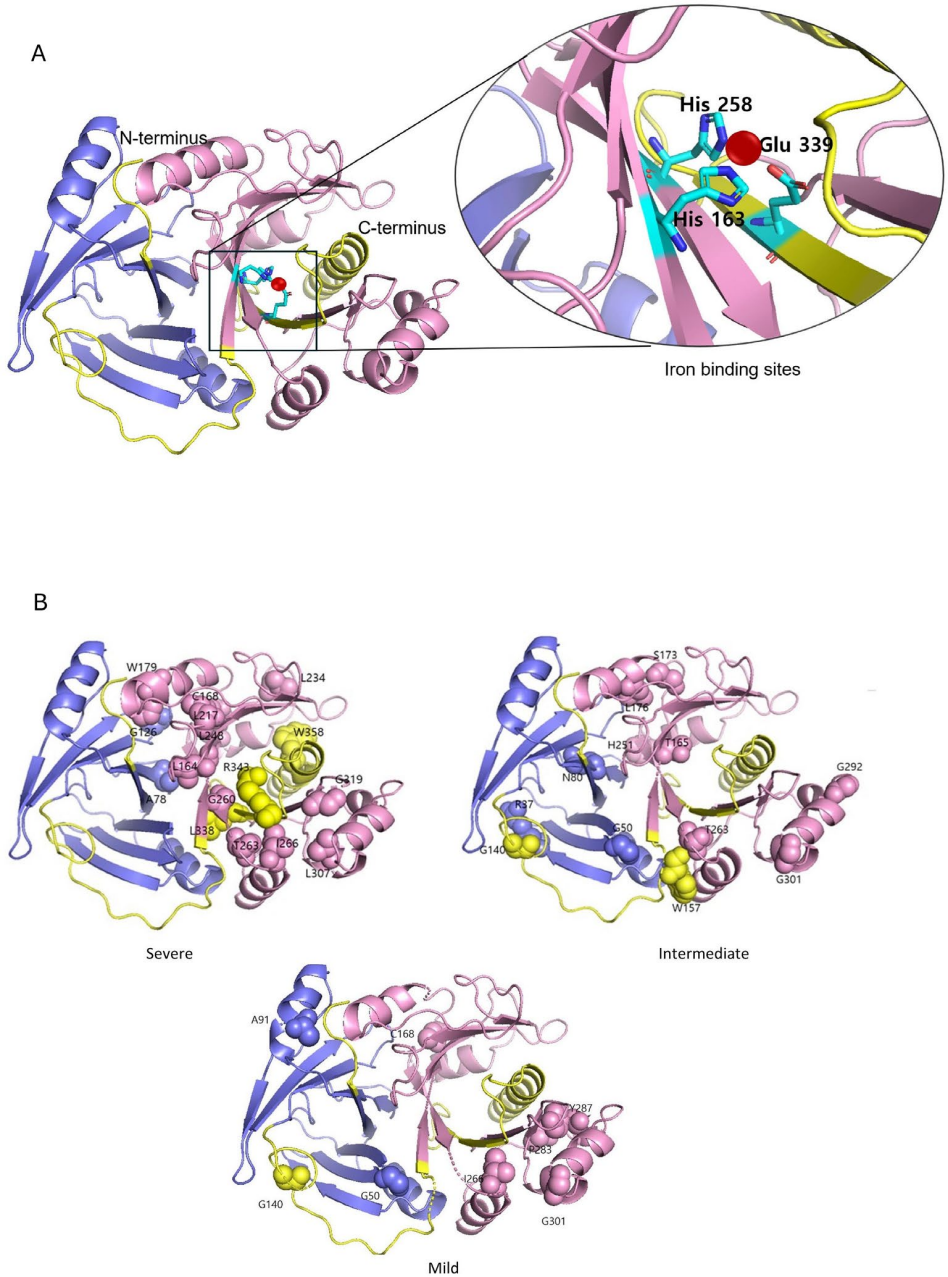
(A) HPDL protein folds to create an iron‐binding pocket. Note that the His 258, His163, and Glu339 residues engage the iron atom (red), involved in oxidase activity. (B) Missense variants in patients with severe phenotypes are predominantly located in or near the VOC2 domain containing iron‐binding site, while missense variants in mild phenotypes are outside the iron‐binding domain. All missense variants in C‐terminus are associated with severe phenotype. Purple: VOC1 domain; Pink: 5 VOC2 domain.

The 3D structural representation using PyMol modeling revealed that missense variants associated with the severe phenotype clustered predominantly in the VOC2 domain, as well as the VOC1 domain close to the VOC2 domain, and the C‐terminal region (Figure 4, Tables S4–S6). Missense variants predicted to be located within 5 Å of the three iron‐binding sites (p.Leu164Pro, p.Gly260Glu, and p.Leu338Pro) were associated with a severe phenotype. Additionally, missense variants located in the C‐terminal region were all associated with a severe phenotype. In contrast, missense variants associated with intermediate and mild phenotypes appeared to be evenly distributed throughout the gene (Table S7).

## 4 Discussion

*HPDL*‐related disease is associated with a broad range of mitochondrial encephalopathy presentations, and previous studies have classified HPDL‐related disease into three main phenotypes, although currently OMIM (Online Mendelian Inheritance in Man) only lists two HPDL phenotypes [3, 5]. Our results concur, suggesting the condition can be better classified into three main phenotypes: severe, intermediate, and mild. The severe phenotype correlates with developmental epileptic encephalopathy, while the mild phenotype correlates with SPG83, with a substantial number of patients falling between the two groups.

The intermediate phenotype was observed in 22 of 99 patients (22.2%), presenting with delayed motor development in late infancy or early childhood. This was distinct from the severe presentation due to a lack of seizures, and from the mild presentation due to a lack of predominant gait disturbance in the second decade. In fact, many intermediate patients were incorrectly diagnosed with idiopathic cerebral palsy before the HPDL diagnosis, initially exhibiting typical development for months to years before experiencing regression. They also demonstrated lower rates of microcephaly, oculomotor abnormalities, and seizures, with less frequent brain MRI abnormalities than in the severe phenotype, also observed in recent reports [3, 9].

Wissner et al. [3] demonstrated that truncating variants tended to be more frequent in subjects with severe phenotypes (47% of alleles) compared to subjects with mild phenotypes (10%), which was further supported by this study. We found that truncating variants associate most closely with the severe phenotype and earlier age of onset. Seventy‐five percent of patients with truncating/truncating variants had a severe phenotype, while none presented with a mild phenotype, in keeping with recent observations [3], and suggesting some residual HPDL activity in intermediate and mild cases.

The p.Met1? pathogenic variant results in the loss of the start codon, which, in a single‐exon gene, is expected to cause loss‐of‐function. Thus, it was unsurprising that this pathogenic variant was associated with the severe phenotype. The common p.Gly50Asp variant was associated with a milder phenotype; however, individuals who were compound heterozygous for p.Gly50Asp experienced disease onset at 2–3 years of age, corresponding to an intermediate phenotype. This suggests that compound heterozygosity for one loss‐of‐function variant may exacerbate the severity, particularly in cases involving the p.Gly50Asp pathogenic variants.

It is notable that 36.4% of missense/missense variants in this study also displayed a severe phenotype with a very early onset age. While studies on residual protein function are still required, nearly all missense variants associated with the severe phenotype are located within or proximal to the VOC2 domain or C‐terminus. The VOC2 domain comprises three iron‐binding residues, likely involved in critical oxidative catalytic functions. There are six proteins in humans containing the VOC domain, all in the metalloenzyme class and sharing a critical βαβββ structural motif (also known as the glyocxylase fold) to build a divalent metal‐containing active site [14]. In the condition methylmalonyl‐CoA epimerase deficiency, enzyme activity in the VOC‐located missense variants was absent [15].

There is clear evidence linking mitochondria to *HPDL*‐related disease, including protein localization to mitochondria and reduction in OXPHOS complex II activity in both HPDL patient‐derived cells and HeLa cells with *HPDL* knockdown [4, 11]. Moreover, the clinical features of *HPDL*‐related disease, such as the broad phenotypic spectrum, neurological dysfunction, and intermittent deterioration, are also consistent with mitochondrial dysfunction. Some patients have bilateral brainstem lesions with increased serum lactate, mimicking Leigh disease [7]. Notably, certain types of HSP including SPG7 and FARS2 are associated with mitochondrial dysfunction. They also exhibit a broad spectrum of diseases, similar to HPDL‐related disorders, including spastic paraplegia and epileptic encephalopathy [16, 17].

HPDL is the sole enzyme known to produce 4‐hydroxymandelic acid (4‐HMA) from 4‐HPPA, a key component in the synthesis of the CoQ10 headgroup. This discovery is significant because it could improve both diagnosis and treatment of *HPDL*‐related disease. 4‐HMA might serve as a biomarker for *HPDL*‐related disease and levels might correlate with clinical severity and the type of pathogenic variant. Although we encountered challenges in measuring 4‐HMA levels in our cohort due to shipping and control issues, further studies are needed to develop biomarkers in *HPDL*‐related disease.

It is notable that 4‐HMA is involved in CoQ10 biosynthesis, which suggests HPDL patients may have low CoQ10 levels. Case 8 in our cohort (Table S1) exhibited a mild reduction in CII + III activity from muscle biopsy, which is CoQ10‐dependent. This finding suggests a possible association between CoQ10 levels and *HPDL*‐related disease and raises the possibility that *HPDL*‐related disease may represent a novel molecular cause of primary CoQ10 deficiency. Primary CoQ10 deficiency refers to the group of conditions characterized by a reduction of CoQ10 levels in tissues or cultured cells associated with a pathogenic variant of the genes involved in the biosynthesis of CoQ10 [18]. Importantly, CoQ10 does not cross the blood–brain barrier well, which limits the effectiveness of direct CoQ10 supplementation for neurological symptoms [19]. Future studies assessing CoQ10 levels in muscle or skin fibroblasts could clarify this relationship.

Along with CoQ10 deficiency as the primary consequence of HPDL deficiency, the potential impact of substrate accumulation upstream of HPDL could, in theory, contribute to disease pathology. However, the specific substrate remains unidentified, and there is no clear evidence of its pathological accumulation. The clinical features of HPDL deficiency are more consistent with CoQ10‐related disorders rather than conditions driven by substrate toxicity. Nevertheless, further studies, including metabolomic profiling, will be necessary to determine whether substrate accumulation plays any role in disease progression.

The discovery of the function of the HPDL enzyme also provided a potential path to treatment for *HPDL*‐related disease, utilizing 4‐HMA or related small molecule therapy. Unlike CoQ10, small molecules like 4‐HMA are expected to cross the blood–brain barrier. Primary CoQ10 deficiency is known to be potentially treatable with high‐dose CoQ10 supplementation in the early stages of the disease; however, neurological symptoms often improve only partially or temporarily [20]. For example, a patient with a homozygous COQ9 [OMIM 614654] pathogenic variant experienced worsening neurological and cardiac symptoms, ultimately passing away at the age of 2 years despite CoQ10 therapy [21]. Another patient with a PDSS2 [OMIM 614651] pathogenic variant developed intractable seizures and died at 8 months old [22]. The response to CoQ10 therapy in patients with cerebellar ataxia has also been variable, with some showing mild clinical improvement while others did not respond [20]. Thus, 4‐HMA and related compounds are promising treatments not just for *HPDL*‐related disease but also for other mitochondrial disorders having a defect in the early stages of the CoQ10 biosynthetic pathway, recently demonstrated for COQ2 deficiency [23]. Ongoing research is being conducted to prepare for a clinical trial of 4‐HMA therapy in *HPDL*‐related disease.

## 5 Online Resource

OMIM: http://omim.org/. ClinVar: https://www.ncbi.nlm.nih.gov/clinvar/. Online registration can be found at: https://neurosciences.ucsd.edu/research/labs/gleeson/hpdl/index.html.

## Author Contributions

E.H.L. and J.G.G. supervised the project and wrote the first draft of the manuscript, which was edited by all authors. O.K.‐M., J.H.Y., and R.H. contributed to clinical outcome measures. M.S.Z., G.M.H.A.‐S., M.S.A.‐H., N.B.‐P., V.M.S., V.K.G., S.G., Y.A., and P.N.T. evaluated patients with *HPDL* variants. Y.N., D.E.‐F., and J.E.A. suggested clinical category definitions. M.Y., L.F., and N.M.O. contributed to clinical coordination and participated in the HPDL clinical trial planning and implementation.

## Conflicts of Interest

None.

## Supporting information


Data S1.

## Data Availability

The data that support the findings of this study are available in the Supporting Information of this article.
