# Correction to “ *HPDL* Variant Type Correlates With Clinical Disease Onset and Severity”

**DOI:** 10.1002/acn3.70084

**Published:** 2025-09-23

E. H. Lee, O. Kim‐Mcmanus, J. H. Yang, et al., “*HPDL* variant type correlates with clinical disease onset and severity,” *Annals of Clinical and Translational Neurology* 12, no. 7 (2025): 1360–1367, https://doi.org/10.1002/acn3.70047.

One of the originally listed authors, Dr. Michael Pacold, has requested to withdraw his authorship, as he believes his contribution does not meet the authorship criteria. The author list and affiliations should be updated to reflect this change by removing his name and affiliation.

We apologize for this error.

