# Supplementary material for: HPDL Variant Type Correlates With Clinical Disease Onset and Severity
Source: Ann Clin Transl Neurol. 2025 May 14;12(7):1360–7. doi: 10.1002/acn3.70047 (PMC12257120; doi:10.1002/acn3.70047)
Supplement: Supplementary file 1 — Data S1. [file ACN3-12-1360-s001.pdf]

Supplementary Table 1. Clinical features of individuals with *HPDL* pathogenic variants

		Case 1	Case 2	Case 3	Case 4	Case 5	Case 6
Country		India	Egypt	Egypt	Egypt	Kuwait	Kuwait
HPDL cDNA variant		C.3G>C	C.928C>T	C.94C>T	C.94C>T	C.94C>T	C.94C>T
HPDL protein variant		P.Met1?	P.Gln310*	P.Gln32*	P.Gln32*	P.Gln32*	P.Gln32*
Zygosity		Homozygous	Homozygous	Homozygous	Homozygous	Homozygous	Homozygous
Gender		F	F	F	F	F	M
Age of onset /current age		15 days/9 months	Birth/deceased at 3 year of age	Birth/6 year 3months	Birth/5 year 3months	3 days/7 year	Birth/deceased at 1months of age
Symptom at onset		Seizures	Weak cry/Poor sucking	Seizures	Seizures	Seizures	Seizures
Severity phenotype		Severe	Severe	Severe	Severe	Severe	Severe
Motor developmental delay		Absent	Absent	Absent	Absent	Absent	NA
ID/GDD		Profound delay	Profound delay	Profound delay	Profound delay	Profound delay	NA
Spasticity		Yes	Yes	Yes	Yes	Yes	No (Hypotonia)
Microcephaly		Yes (-3.9SD at 9mo)	Yes (-3.2SD at 3 years)	Yes	Yes	Yes	N.A
Seizure/type		Yes /infantile spasm	Yes /focal seizures	Yes/tonic, myoclonic	Yes/tonic, myoclonic	Yes/GTC, focal	Yes/GTC, tonic, focal
Encephalopathic episodes		No	No	No	No	No	No
Oculomotor abnormalities		Nystagmus	Yes	Nystagmus	Nystagmus	No	NA
Dysphagia		Yes	Yes	Yes	Yes	Yes	NA
Ataxia/tremor		Yes/No	No/No	NA/Yes	NA/Yes	NA/Yes	NA
Brain MRI	Cortical atrophy	No	No	Yes	Yes	Yes	NA
	CC hypoplasia	Yes	Yes	Yes	Yes	Yes	NA
	WM defects	Yes	Yes	Yes	Yes	Yes	NA
	Cerebellar hypoplasia	Yes	Yes	Yes	Yes	Yes	NA
MRS		ND	ND	ND	ND	ND	ND
Respiratory enzyme analysis		ND	ND	ND	ND	ND	ND

		Case 7	Case 8	Case 9	Case 10	Case 11	Case 12	Case 13
Country		Egypt	Italy	USA	USA	Sudan	Turkey	Germany
HPDL cDNA variant		c.436C>G	c.1058A>T;c.1-?_1116+?del	c.3G>C;c.789del	c.238A>G	c.875G>A	c.149G>A	c.149G>A
HPDL protein variant		p.Phe146Val	p.Asn353Ile;p.?	p.Met1?;p.Pro264Leufs*51	p.Asn80Asp	p.Gly292Glu	p.Gly50Asp	p.Gly50Asp
Zygosity		Homozygous	Compound heterozygous	Compound heterozygous	Homozygous	Homozygous	Homozygous	Homozygous
Gender		F	F	M	M	F	M	M
Age of onset/current age		4-5 months/8 year 6 months	2 months/13 years	8 years/8 years	12 months/8 years	3 year 2 months/ 6 year 7 months	11 year/17 year	8 years/14 years
Symptoms at onset		Developmental delay	Seizures	Gait problem	Developmental delay	Developmental regression	Gait problem	Gait problem
Phenotype		Severe	Severe	Intermediate	Intermediate	Intermediate	Mild	Mild
Motor developmental delay		Delayed	Delayed	Normal, rapid regression	Delayed	Normal development then regressed	Normal	Normal
ID/GDD		Profound delay	Delayed	Normal	Delayed	Mild to moderate ID	Normal	Normal
Spasticity		Yes	Yes	Yes	Yes	Yes	Yes	Yes
Microcephaly		Yes	Yes	No	No	No	No	No
Seizure/type		Yes/tonic, myoclonic	Yes /generalized tonic-clonic	No	No	Yes/tonic	No	No
Encephalopathic episodes		No	No	No	No	No	No	No
Oculomotor abnormalities		No	No	No	Nystagmus	No	No	No
Dysphagia		No	No	No	No	Yes/NA	No	No
Ataxia/tremor		No/Yes	No/No	Yes/Yes	No/NA	Yes/No	Yes/NA	No/No
Brain MRI	Cortical atrophy	Yes	Yes	No	No	Yes	Normal	Normal
	CC hypoplasia	Yes	Yes	No	Yes	Yes	Normal	Normal
	WM defects	Yes	Yes	No	Yes	No	Normal	Normal
	Cerebellar hypoplasia	Yes	No	No	No	Yes	Normal	Normal
MRS		ND	Increased NAA	ND	ND	ND	ND	ND
Respiratory enzyme analysis		ND	Mild reduction in complex II and complex II+III activities.	ND	ND	ND	ND	ND

Abbreviations: F: female; M: male; ID: Intellectual disability; GDD: Global developmental delay; MRI: Magnetic resonance imaging; CC: Corpus callosum; WM: White matter; MRS: Magnetic resonance spectroscopy; NAA: N-acetylaspartate; NA: Not Available; ND: Not done; SD: Standard deviation

Supplementary Table 2. Clinical manifestations in patients with c.149G>C (p.Gly50Asp) homozygous genotype

Gender	Country	Severity	Onset age (years)	Initial symptoms	Last examination (years)	Motor development	Seizures	Neuroimaging	Higher cognitive functions	References
F	Sudan	Mild	NA	NA	11	Delayed	No	Normal	Mild ID	3
F	Syria	Intermediate	3	Gait problem	16	Normal	No	Cerebellar atrophy	Normal	4
M	Syria	Mild	12	Gait problem	14	Normal	No	ND	Normal	4
M	Turkey	Mild	13	Gait problem	17	Normal	No	Normal	Normal	4
M	Egypt	Mild	15	LL weakness, progressive	17	Delayed	No	Normal	Delayed	4
M	Egypt	Mild	14	Gait problems, LL stiffness, frequent falls	16	Delayed	NA	ND	Delayed	4
F	Egypt	Mild	11	LL stiffness, frequent falls	13	Normal	NA	Normal	Normal	4
M	Syria	Mild	12	LL stiffness and pain	15	Normal	No	Normal	Normal	5
F	Turkey	Mild	15	Gait instability, progression	20	Normal	No	Normal	Normal	5
M	Syria	Mild	15	Gait instability	19	Normal	No	Normal	Normal	5
M	Turkey	Mild	14	Weakness, progression spasticity	39	Normal	No	ND	Normal	5
M	Turkey	Mild	15	Gait problem	33	Normal	No	NA	Normal	5
M	Germany	Mild	8	Gait problem	14	Normal	No	Normal	Normal	Our cohort
M	Turkey	Mild	11	Gait problem	17	Normal	No	Normal	Normal	Our cohort

Abbreviations: NA: Not available; ID: Intellectual disability; LL: Lower legs; ND: Not done

Supplementary Table 3. Truncating/truncating variants are associated with severe phenotype.

Severity group	Missense Missense	Missense Truncating	Truncating Truncating	Total
Mild	17 (30.9%)	5 (31.3%)	0 (0%)	
Intermediate	18 (32.7%)	4 (25.0%)	7 (25.0%)	
Severe	20 (36.4%)	7 (43.8%)	21 (75.0%)	
Total	55	16	28	99

p=0.005 (Chi-square test)

Supplementary Table 4. Missense/missense variants with severe phenotypes

**Allele 1**	**Allele 2**	**Number of patients**	**Zygosity**	**Location of the mutation**
p.Ala78Thr	p.Ala78Thr	3	Homozygous	VOC1domain
p.Gly126Ser	p.Gly126Ser	1	Homozygous	VOC1 domain
p.Leu164Pro*	p.Leu164Pro*	2	Homozygous	VOC2 domain
P.Leu217Pro	P.Leu217Pro	1	Homozygous	VOC2 domain
p.Gly260Glu*	p.Gly260Glu*	2	Homozygous	VOC2 domain
p.Thr263Met	p.Thr263Met	1	Homozygous	VOC2 domain
p.Thr263Arg	p.Thr263Arg	1	Homozygous	VOC2 domain
p. Leu338Pro*	p. Leu338Pro*	1	Homozygous	C-terminal
p.Trp358Gly	p.Trp358Gly	3	Homozygous	C-terminal
p.Cys168Tyr	p.Trp179Cys	2	Compound Heterozygous	VOC2 domain VOC 2 domain
p.Leu217Pro	p.Ile266Thr	1	Compound Heterozygous	VOC2 domain VOC2 domain
p.Leu234Pro	p.Leu248Pro	1	Compound Heterozygous	VOC2 domain VOC2 domain
p. Arg343Ser	p.Leu307Pro	1	Compound Heterozygous	C-terminal VOC2 domain

* Variants within 5 Å of iron binding site

VOC:vicinal oxygen chelate

Supplementary Table 5. Missense/missense variants with intermediate phenotypes

**Allele1**	**Allele 2**	**Number of patients**	**Zygosity**	**Location of the mutation**
p.Arg37Pro	p.Arg37Pro	1	Homozygous	VOC1 domain
pGly50Asp	pGly50Asp	1	Homozygous	VOC1 domain
p.Asn80Asp	p.Asn80Asp	1	Homozygous	VOC1 domain
p.Gly140Arg	p.Gly140Arg	3	Homozygous	Between the two domains
p.Thr165Pro*	p.Thr165Pro*	1	Homozygous	VOC2 domain
p.Leu176Pro	p.Leu176Pro	7	Homozygous	VOC2 domain
p. Gly292Glu	p. Gly292Glu	1	Homozygous	VOC2 domain
p.Gly301Val	p.Gly301Val	1	Homozygous	VOC2 domain
p.Ser173Tyr	p.Thr263Arg	1	Compound Heterozygous	VOC2 domain<br>VOC2 domain
p.Trp157Arg	p.His251Gln	1	Compound Heterozygous	VOC2 domain<br>VOC2 domain

* Variants within 5 Å of iron binding site

Supplementary Table 6. Missense/missense variants with mild phenotypes

**Allele1**	**Allele 2**	**Number of patients**	**Zygosity**	**Location of the mutation**
p.Gly50Asp	p.Gly50Asp	14	Homozygous	VOC1 domain
p.Gly301Val	p.Gly301Val	1	Homozygous	VOC2 domain
p.Tyr287His	p.Pro283Ser	1	Compound Heterozygous	VOC2 domain<br>VOC2 domain
p.Ile266Thr	p.Cys168Tyr	1	Compound Heterozygous	VOC2 domain<br>VOC2 domain
p.Ala91Pro	p.Gly140Gly	1	Compound Heterozygous	VOC1 domain<br>VOC2 domain

Supplementary Table 7. In silico analysis of the effects of *HPDL* missense variants

Location	cDNA	AA Change	CDS position	Protein position	Amino acids	SIFT	PolyPhen	gnomADe AF	CADD PHRED	CADD RAW	Mutation Taster score	PROVEAN score	REVEL score
1:45792930-45792930	c.110G>C	p.Arg37Pro	110	37	R/P	Deleterious(0)	Possibly_damaging (0.491)	4.367e-06	27.6	3.990217	0.999997	-2.07	
1:45792951-45792951	c.131A>T	p.Gln44Leu	131	44	Q/L	Deleterious(0)	Possibly_damaging (0.549)	0	25.6	3.655408	0.999957	-3.61	0.326
1:45792969-45792969	c.149G>A	p.Gly50Asp	149	50	G/D	Deleterious(0)	Probably_damaging (0.931)	1.018e-05	25.1	3.520986	0.999977	-4.72	0.586
1:45793052-45793052	c.232G>A	p.Ala78Thr	232	78	A/T	Deleterious(0.01)	Probably_damaging (0.925)	0	28.2	4.063582	0.999864	-0.84	0.289
1:45793058-45793058	c.238A>G	p.Asn80Asp	238	80	A/G	Deleterious(0.01)	Benign (0.116)		24.2	3.244274	0.999969	1.12	0.245
1:45793091-45793091	c.271G>C	p.Ala91Pro	271	91	A/P	Tolerated(0.14)	Possibly_damaging (0.812)		21.9	2.273832	0.999959	-3.19	0.402
1:45793196-45793196	c.376G>A	p.Gly126Ser	376	126	G/S	Deleterious(0.02)	Probably_damaging (0.999)	8.669e-06	25.1	3.539462	1	-3.43	0.639
1:45793238-45793238	c.418G>A	p.Gly140Arg	418	140	G/R	Deleterious(0)	Possibly_damaging (0.706)		25.1	3.523752	0.999999	-7.22	0.629
1:45793239-45793239	c.419G>A	p.Gly140Glu	419	140	G/E	Deleterious(0)	Possibly_damaging (0.793)		24.4	3.322445	0.999994	-7.07	0.544
1:45793256-45793256	c.436C>G	p.Phe146Val	436	146	F/V	Deleterious(1)	Probably_damaging (0.991)		23.7	3.048459	0.999996	-5.3	0.576
1:45793289-45793289	c.469T>C	p.Trp157Arg	469	157	W/R	Tolerated(0.49)	Benign (0.006)	4.61e-05	19.94	2.0257	1	1.8	0.193
1:45793311-45793311	c.491T>C	p.Leu164Pro	491	164	L/P	Deleterious(0)	Probably_damaging (0.935)		29.8	4.241212	1	-3.92	0.616
1:45793313-45793313	c.493A>C	p.Thr165Pro	493	165	T/P	Deleterious(0)	Probably_damaging (0.953)	4.124e-06	25.8	3.708628	0.9969	-3.21	0.576
1:45793323-45793323	c.503G>A	p.Cys168Tyr	503	168	C/Y	Deleterious(0)	Probably_damaging	8.246e-06	29.8	4.246169	1	-4.29	0.611

							(0.984)						
1:45793338-45793338	c.518C>A	p.Ser173Tyr	518	173	S/Y	Deleterious(0)	Probably_damaging (0.964)		27.7	3.99762	0.992959	-2.1	0.32
1:45793347-45793347	c.527T>C	p.Leu176Pro	527	176	L/P	Deleterious(0.01)	Possibly_damaging (0.601)	4.086e-06	23.8	3.102843	0.999711	-3.05	0.38
1:45793357-45793357	c.537G>C	p.Trp179Cys	650	217	L/P	Deleterious(0)	Probably_damaging (0.983)		25.3	3.597968	1	-1.51	0.555
1:45793389-45793389	c.569C>T	p.Pro190Leu	569	190	P/L	Tolerated(0.92)	Benign(0.01)	4.038e-06	21.8	2.256191	0.99986	1.75	0.073
1:45793470-45793470	c.650T>C	p.Leu217Pro	650	217	L/P	Deleterious(0)	Probably_damaging (0.983)		25.3	3.597968	1	-1.51	0.555
1:45793512-45793512	c.692C>G	p.Ala231Gly	692	231	A/G	Deleterious(0.02)	Possibly_damaging (0.703)		23.6	2.998027	0.999895	-1.29	0.243
1:45793521-45793521	c.701T>C	p.Leu234Pro	701	234	L/P	Deleterious(0)	Probably_damaging (0.99)	5.019e-06	24.1	3.206391	0.999961	-3.21	0.453
1:45793563-45793563	c.743T>C	p.Leu248Pro	743	248	L/P	Deleterious(0)	Probably_damaging (0.998)		29.8	4.239637	1	-6.56	0.798
1:45793573-45793573	c.753C>A	p.His251Gln	753	251	H/Q	Deleterious(0)	Possibly_damaging (0.831)		23.4	2.924623	0.999821	-4.87	0.831
1:45793599-45793599	c.779G>A	p.Gly260Glu	779	260	G/E	Deleterious(0.01)	Probably_damaging (0.978)	4.509e-06	24.4	3.326313	0.999899	-3.91	0.613
1:45793601-45793601	c.781C>G	p.Leu261Val	781	261	L/V	Deleterious(0)	Probably_damaging (0.991)		23.4	2.940846	0.999979	-2.45	0.508
1:45793608-45793608	c.788C>G	p.Thr263Arg	788	263	T/R	Deleterious(0)	Probably_damaging (0.999)		25.4	3.617869	1	-5.73	0.741
1:45793608-45793608	c.788C>T	p.Thr263Met	788	263	T/M	Deleterious(0)	Probably_damaging (0.998)	1.306e-05	26	3.753706	1	-5.46	0.715
1:45793617-45793617	c.797T>C	p.Ile266Thr	797	266	I/T	Deleterious(0)	Probably_damaging (0.998)	8.506e-06	23.9	3.151197	1	-4.85	0.813
1:45793667-45793667	c.847C>T	p.Pro283Ser	847	283	P/S	Deleterious(0.01)	Probably_damaging (0.944)	4.793e-05	23.7	3.067566	0.99692	-2.21	0.28

1:45793679-45793679	c.859T>C	p.Tyr287His	859	287	Y/H	Deleterious(0)	Probably_damaging (1)	9.954e-05	25.5	3.636008	0.999999	-4.91	0.882
1:45793695-45793695	c.875G>A	p.Gly292Glu	875	292	G/E	Deleterious(0.04)	Benign(0.143)		23.1	2.756787	0.982102	-0.06	0.162
1:45793722-45793722	c.902G>T	p.Gly301Val	902	301	G/V	Deleterious(0)	Probably_damaging (0.983)		24.6	3.375162	1	-2.86	0.412
1:45793833-45793833	c.1013T>C	p.Leu338Pro	1013	338	L/P	Deleterious(0)	Probably_damaging (0.993)	2.387e-05	29.9	4.263384	1	-4.98	0.761
1:45793878-45793878	c.1058A>T	p.Asn353Ile	1058	353	N/I	Deleterious(0)	Probably_damaging (1)		28.9	4.137769	1	-8.69	0.884
1:45793892-45793892	c.1072T>G	p.Trp358Gly	1072	358	W/G	Deleterious(0)	Probably_damaging (0.972)		29	4.149109	0.999999	-7.67	0.638
